# Cost-effectiveness of durvalumab plus tremelimumab in combination with chemotherapy for the treatment of metastatic non-small-cell lung cancer from the US healthcare sector’s and societal perspectives

**DOI:** 10.3389/fphar.2024.1256992

**Published:** 2024-06-10

**Authors:** Yena Gan, Fenghao Shi, He Zhu, Huangqianyu Li, Sheng Han, Duoduo Li

**Affiliations:** ^1^ Dongzhimen Hospital, Beijing University of Chinese Medicine, Beijing, China; ^2^ International Research Center for Medicinal Administration, Peking University, Beijing, China; ^3^ School of Pharmaceutical Sciences, Peking University, Beijing, China

**Keywords:** NSCLC, durvalumab, tremelimumab, healthcare, cost-effectiveness

## Abstract

**Purpose:**

Metastatic non-small cell lung cancer (mNSCLC) has a high incidence rate, and economic burdens to patients, healthcare systems, and societies. Durvalumab plus tremelimumab and chemotherapy (T+D+CT) is a novel therapeutic strategy for mNSCLC, which demonstrated promising efficacy in a phase-3 randomized clinical trial, but its economic value remains unclear.

**Methods:**

This economic evaluation used a hypothetical cohort of patients with mNSCLC, with characteristics mirroring those of the participants in the POSEIDON trial. Several partitioned survival models were constructed to estimate 15-year costs and health outcomes associated with the T+D+CT, durvalumab plus chemotherapy (D+CT) and chemotherapy alone (CT) strategies, discounting costs and effectiveness at 3% annually. Costs were in 2023 US dollars. Data were derived from the POSEIDON trial and published literature. Deterministic and probabilistic sensitivity analyses were performed to assess the uncertainty of input parameters and study generalizability. The analysis was designed and conducted from September 2022 to March 2023. To evaluate the cost-effectiveness of T+D+CT, compared with CT and D+CT, for mNSCLC from the perspectives of the US healthcare sector and society.

**Findings:**

From the healthcare sector’s perspective, the T+D+CT yielded an additional 0.09 QALYs at an increased cost of $7,108 compared with CT, which resulted in an ICER of $82,501/QALY. The T+D+CT strategy yielded an additional 0.02 QALYs at an increased cost of $27,779 compared with the D+CT, which resulted in an ICER of $1,243,868/QALY. The economic results of T+D+CT vs. CT were most sensitive to the annual discount rate, subsequent immunotherapy cost, tremelimumab cost, palliative care and death cost, pemetrexed cost, and durvalumab cost. The T+D+CT strategy was considered cost-effective relative to CT in 59%–82% of model iterations against willingness-to-pay. thresholds of $100,000/QALY gained to $150,000/QALY gained. From the societal perspective, the T+D+CT can be considered as cost-effective as compared with CT or D+CT, independent of histology.

**Implications:**

In this cost-effectiveness analysis, the T+D+CT strategy represented good value compared with CT for patients with mNSCLC from the perspectives of the healthcare sector and the society. This treatment strategy may be prioritized for mNSCLC patients at high risks of disease progression.

## 1 Introduction

Non-small cell lung cancer (NSCLC) continues to be the leading cause of cancer-related mortality worldwide (Bray et al., 2018; Howlader et al., 2020; Siegel et al., 2022). Approximately one-half of patients have advanced or metastatic stage III disease at the time of diagnosis and many patients with local or regional disease subsequently develop recurrent or metastatic disease, the prognosis for which has been poor, with a five-year survival of approximately 9% (American Cancer Society, 2022). Immune checkpoint inhibitors targeting programmed cell death ligand-1/programmed cell death-1 (PD-L1/PD-1) have significantly improved patient outcomes and become the standard of care for metastatic NSCLC (mNSCLC) (Gandhi et al., 2018; Socinski et al., 2018).

Pembrolizumab, a selective, high-affinity human IgG1 monoclonal antibody (mAb) that blocks PD-L1 binding to PD-1 and CD80, is approved for the first-line monotherapy of patients with PD-L1-positive (tumor proportion score of 1% or more) tumors in the US (Azpicentral, 2022). Tremelimumab, a selective human IgG2 mAb that blocks cytotoxic T-lymphocyte associated protein 4 (CTLA-4) binding to B7.1 and B7.2 ligands, is approved for the treatment of patients with mNSCLC in combination with durvalumab and platinum-based chemotherapy in the US(Keam, 2023). POSEIDON (a phase III, global, randomized, open-label trial of tremelimumab plus durvalumab and chemotherapy (T+D+CT) or durvalumab plus chemotherapy (D+CT) vs. chemotherapy alone (CT) in patients with mNSCLC; [ClinicalTrials.gov identifier: NCT03164616]) clinical trial recently found that the combination of two immune checkpoint inhibitors, tremelimumab plus durvalumab (alongside chemotherapy), as the first-line treatment improved overall survival (OS) and progression-free survival (PFS) in patients with mNSCLC compared with CT, independent of PD-L1 expression (Johnson et al., 2023). It also found T+D+CT to be more efficacious than D+CT. However, T+D+CT resulted in a higher rate of treatment-related adverse events (TRAEs) than CT and D+CT.

Although T+D+CT showed promising results in treating mNSCLC, it remains unknown whether T+D+CT entails longer-term economic benefits. With the incidence rate of mNSCLC increasing and launch of highly priced anticancer agents, healthcare expenditure on novel anticancer treatments is rapidly expanding (Planchard et al., 2018; Chen et al., 2020; Horvath et al., 2020; Kasahun et al., 2020; Diao et al., 2022). This not only entails economic burden in itself but also can lead to compromised patient outcomes such as decreased quality of life (QoL) of patients who quit or delay treatment due to financial concerns (Courtney et al., 2021). This necessitates assessment of the cost-effectiveness of novel treatment regimens. In this study, we conducted a computer simulation model to assess the cost-effectiveness of T+D+CT compared with CT and D+CT as first-line treatment for patients with mNSCLC from the US healthcare sector and societal perspectives (Planchard et al., 2018; Chen et al., 2020; Horvath et al., 2020; Kasahun et al., 2020; Diao et al., 2022).

## 2 Methods

This economic evaluation used published clinical trial data and was therefore deemed exempt from institutional review board approval and informed consent by the institutional review board of Peking University, China. Economic analyses complied with the methodological guidelines set by the US Second Panel on Cost-Effectiveness in Health and Medicine and were reported in compliance with the Consolidated Health Economic Evaluation Reporting Standards 2022 (CHEERS) checklist (CHEERS, 2022).

### 2.1 Decision model

**FIGURE 1:**
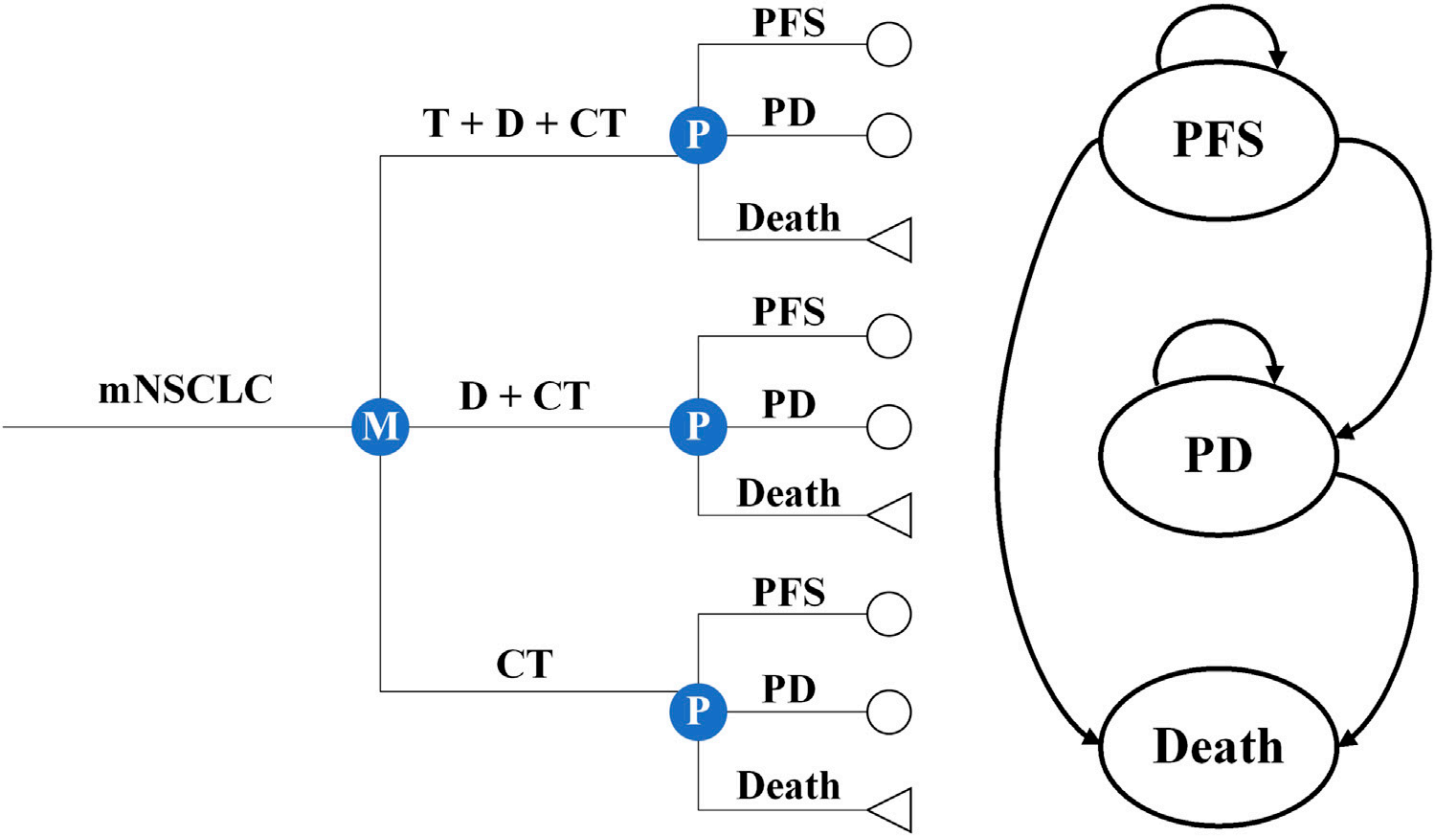
Microsimulation and decision tree model for different treatment regimens and health states. mNSCLC, metastatic non-small cell lung cancer; T+D+CT, tremelimumab plus durvalumab and chemotherapy; D+CT, durvalumab plus chemotherapy; CT, chemotherapy alone; PFS, progression-free disease; PD, progressive disease.

We constructed several partitioned survival models to simulate the cost-effectiveness of T+D+CT vs. CT and D+CT as the first-line therapy for mNSCLC patients from the perspectives of the US healthcare sector and the society. These models were constructed with a one-month cycle length and a horizon extending over 15 years, including three mutually exclusive health states: PFS, progressive disease (PD), and death (Figure 1). We constructed a hypothetical cohort of patients who had characteristics consistent with those of the participants in the POSEIDON clinical trial (Supplementary eMethods). Patients entered the model in the PFS state; they could then remain in this state or experience TRAEs, PD, or death. The primary outcomes of the models were the direct costs associated with mNSCLC treatment and management and quality-adjusted life years (QALYs), which were used to derive the incremental cost-effectiveness ratio (ICER) and then compared with the willingness-to-pay (WTP) threshold of $100,000/QALY (Neumann et al., 2014). Both costs and QALYs were discounted at 3% annually (Sanders et al., 2016). All monetary terms were converted to 2023 US dollars using the Consumer Price Index. The Excel spreadsheet software (version 16, Microsoft) was used to build and run models. Data analyses were conducted from September 2022 to March 2023.

### 2.2 Treatment details

The POSEIDON clinical trial stratified patients by PD-L1 expression (tumor proportion score ≥50% or <50%) and randomized patients to receive T+D+CT, D+CT, or CT (Supplementary eMethods). All clinical data used in our primary cost-effectiveness analysis were obtained from the POSEIDON clinical trial. The base case models followed the POSEIDON trial protocol, in which patients received treatment with durvalumab or durvalumab-tremelimumab combination therapy until PD or unacceptable TRAEs, whichever occurred first. Per the POSEIDON protocol, certain patients could continue to receive durvalumab monotherapy after PD if they continued to receive benefit and met prespecified criteria. For patients who received five cycles of durvalumab-tremelimumab combination therapy and subsequently had PD during durvalumab monotherapy, they could receive retreatment with up to four additional cycles of tremelimumab alongside durvalumab. In accordance with the POSEIDON protocol, patients who were receiving upfront chemotherapy in our base case models also received treatment until PD, unacceptable TRAEs, or 18 weeks of treatment, whichever occurred first. In addition, per the POSEIDON protocol, patients with non-squamous histology who received cisplatin or carboplatin plus pemetrexed could receive pemetrexed maintenance therapy until PD or unacceptable TRAEs.

### 2.3 Model parameters

#### 2.3.1 Model parameters

The probabilities for the partitioned states were derived from the reported Kaplan-Meier (K-M) curves of OS and PFS in the POSEIDON (Supplementary eMethods; Supplementary eFigure S1; Supplementary eTable S1). Of note, the trial only reported PFS data through 23 months and OS data through 44 months after the initiation of treatment. The following survivals were estimated using parametric survival functions (Supplementary eMethods; Supplementary eFigure S1).

#### 2.3.2 Costs

We considered costs from both the healthcare sector’s and societal perspectives. The formal healthcare costs consisted of costs attributable to drugs, management of TRAEs, imaging, best supportive care (BSC), radiotherapy, and palliative care and death. Drug costs were extracted from the literature (Tringale et al., 2018; Wu et al., 2018), the reimbursement schedule shown by the Centers for Medicare and Medicaid Services (CMS) (Centers for Medicare, 2023), or average wholesale price (AWP) (Pemetrexed, 2022; Tremelimumab, 2022; Durvalumab, 2023) and were then calculated by summing the drug’s AWP plus costs of infusion and follow-up and monitoring (Tringale et al., 2018; Wu et al., 2018). Costs to manage TRAEs were included as a weighted average based on the number of reported severe TRAEs (grades 3/4) in the clinical trial (Johnson et al., 2023). Costs of imaging, BSC, radiotherapy, and palliative care and death were obtained from the literature (Sher et al., 2011; Criss et al., 2019; Insinga et al., 2019). The model to depict the societal perspective incorporated informal healthcare costs (patient time and/or salary, transportation, and caregiver costs) (Lauzier et al., 2011; Li et al., 2013; Guérin et al., 2016) and non-healthcare costs (productivity loss) (Guérin et al., 2016).

#### 2.3.3 Health utilities

Health utility was measured on a scale of 0–1, with 1 corresponding to optimal health and 0 corresponding to death; specific values in this study were obtained from published literature (Sanders et al., 2016). A decrement in health utility was known as disutility and occurred when experiencing TRAEs. Disutility associated with specific TRAEs were extended over a cycle period and their weighted averages were calculated paralleling their frequency in the POSEIDON clinical trial (Table 1). A weighted aggregate of health utilities overtime was used to measure QALYs, which reflected treatment effectiveness.

**TABLE 1:** Model parameters.

Parameter	Base-case value (range)	Distribution	Source
Cost			
Treatment cost ($/cycle)			
Durvalumab	1,380 (1,104–1,656)	Gamma	Durvalumab (2023)
Tremelimumab	9,360 (7,488–11,232)	Gamma	Tremelimumab (2022)
Abraxane	6,395 (5,116–7,674)	Gamma	Centers for Medicare (2023)
Pemetrexed	2,117 (1,693–2,540)	Gamma	Pemetrexed (2022)
Gemcitabine	78 (62–94)	Gamma	Centers for Medicare (2023)
Platinum doublet	12 (0.37–27)	Gamma	Centers for Medicare (2023)
Subsequent immunotherapy	12,592 (7,757–17,281)	Gamma	Centers for Medicare (2023)
Docetaxel	77 (62–92)	Gamma	Centers for Medicare (2023)
Radiotherapy	279 (223, 335)	Gamma	Centers for Medicare (2023)
Imaging	1,409 (1,127, 1,691)	Gamma	Criss et al. (2019)
BSC	637 (510–764)	Gamma	Criss et al. (2019)
Palliative care and death	15,957 (12,766, 19,148)	Gamma	Insinga et al. (2019)
Administration cost ($/cycle)			
Drug administration per hour	143 (114, 172)	Gamma	Criss et al. (2019)
Follow-up and monitoring	433 (346, 520)	Gamma	Insinga et al. (2019)
Cost to manageTRAEs ($/event)			
Anemia	5,243 (4,195, 6,292)	Gamma	Smith et al. (2002)
Neutropenia	16,857 (13,486, 20,229)	Gamma	Hornberger et al. (2015)
Thrombocytopenia	836 (669, 1,003)	Gamma	Insinga et al. (2019)
Neutrophil count decreased	907 (726, 1,088)	Gamma	Insinga et al. (2019)
Societal costs			
Patient time and salary loss ($/cycle)	550 (440, 660)	Gamma	Guérin et al. (2016)
Parking, meals, and travel ($/time)	33 (27, 40)	Gamma	Lauzier et al. (2011)
Caregiver ($/cycle)	640 (512, 768)	Gamma	Li et al. (2013)
Productivity loss ($/cycle)	881 (705, 1,057)	Gamma	Guérin et al. (2016)
Health utilities			
Disease status utility per year			
mNSCLC			
PFS	0.82 (0.65, 0.98)	Beta	Grutters et al. (2010)
PD	0.32 (0.26, 0.39)	Beta	Nafees et al. (2017)
Nonsquamous mNSCLC			
PFS	0.84 (0.67, 0.88)	Beta	Nafees et al. (2017)
PD	0.47 (0.17, 0.57)	Beta	Nafees et al. (2008)
Squamous mNSCLC			
PFS	0.71 (0.67, 0.76)	Beta	Chouaid et al. (2013)
PD	0.18 (0.14, 0.22)	Beta	Nafees et al. (2008)
TRAEs disutility per year			
Anemia	0.06 (0.05, 0.07)	Beta	Freeman et al. (2015)
Neutropenia	0.03 (0.02, 0.03)	Beta	Johnson et al. (2023)
Thrombocytopenia	0.11 (0.09, 0.13)	Beta	Tolley et al. (2013)
Neutrophil count decreased	0.03 (0.02, 0.03)	Beta	Hornberger et al. (2015)
Risk of TRAEs (%, rate of grade 3/4 over 5%)			
T+D+CT			
Anemia	17.27 (13.82, 20.73)	Beta	Johnson et al. (2023)
Neutropenia	16.06 (12.85, 19.27)	Beta	Johnson et al. (2023)
Thrombocytopenia	5.45 (4.36, 6.55)	Beta	Johnson et al. (2023)
Neutrophil count decreased	7.27 (5.82, 8.73)	Beta	Johnson et al. (2023)
D+CT			
Anemia	15.27 (12.22, 18.32)	Beta	Johnson et al. (2023)
Neutropenia	12.57 (10.06, 15.09)	Beta	Johnson et al. (2023)
Thrombocytopenia	4.49 (3.59, 5.39)	Beta	Johnson et al. (2023)
Neutrophil count decreased	7.19 (5.75, 8.62)	Beta	Johnson et al. (2023)
CT			
Anemia	2.04 (16.34, 24.50)	Beta	Johnson et al. (2023)
Neutropenia	12.01 (9.61, 14.41)	Beta	Johnson et al. (2023)
Thrombocytopenia	5.11 (4.08, 6.13)	Beta	Johnson et al. (2023)
Neutrophil count decreased	7.51 (6.01, 9.01)	Beta	Johnson et al. (2023)
Annual discount rate (%)	3 (1, 5)	Beta	Murray et al. (2000)

BSC, best supportive care; TRAEs, treatment-related adverse events; mNSCLC, metastatic non-small cell lung cancer; PFS, progression-free disease; PD, progressive disease; T+D+CT, tremelimumab plus durvalumab and chemotherapy; D+CT, durvalumab plus chemotherapy; CT, chemotherapy alone.

### 2.4 Statistical analysis

#### 2.4.1 Cost-effectiveness analysis

The ICERs of T+D+CT vs. CT and T+D+CT vs. D+CT were used to assess the cost-effectiveness, which were measured using the incremental total healthcare or social costs divided by the incremental total QALYs. Treatment was considered cost-effective when the ICER was less than the WTP of $100,000/QALY (Neumann et al., 2014). The ICERs were rounded to the nearest $100,000. The impact inventory for the parameters considered in economic analyses was provided in Table 1.

#### 2.4.2 Sensitivity analysis

One-way deterministic sensitivity analyses (OWSAs) and probabilistic sensitivity analyses (PSAs) were performed to assess the impact of parameter uncertainties on ICERs. In the sensitivity analysis, costs were modeled with gamma distributions, and health utilities, transition probabilities, and rates of TRAEs and discount were modeled with beta distributions. Standard deviations (SDs) for each distribution were obtained from the literature when possible. Unknown SDs were calculated using 20% of the mean. PSAs simulated 10,000 variations of all model parameters. In addition, we analyzed the expected value of perfect information (EVPI) to evaluate uncertainty in allocating treatment to the appropriate patients who might benefit in the most cost-effective manner.

#### 2.4.3 Scenario analysis

Patients who still adhered to the treatments in the trial at the final data collection point (24 July 2019) were included in the scenario analysis. Assuming these patients had been cured, they discontinued the aforementioned therapies but were still followed up monthly until 15 years. The survival data followed the age-adjusted survival probabilities of the general US population provided by actuarial life tables from the US Social Security Administration (Courtney et al., 2021).

#### 2.4.4 Subgroup analysis

In POSEIDON, patients with squamous histology receiving T+D+CT benefited less in PFS and OS than those with non-squamous histology, even if they experienced improved benefits compared with the CheckMate 227 trial (another clinical trial that observed CTLA-4 plus PD-L1 and chemotherapy for mNSCLC) (Hellmann et al., 2019; Johnson et al., 2023). Therefore, subgroup analysis was conducted to explore possible heterogeneity between patients with non-squamous mNSCLC and squamous mNSCLC.

## 3 Results

### 3.1 Base case analysis

From the perspective of the US healthcare, T+D+CT was associated with an increased cost of $7,108 from $360,968 for CT and an increased cost of $27,779 from $340,297 for D+CT. Treatment with T+D+CT yielded a gain of 0.09 QALYs from 0.46 QALYs for CT and a gain of 0.02 QALYs from 0.53 QALYs for D+CT, resulting in ICERs of $82,501/QALY for CT and $1,243,868/QALY for D+CT. From the societal perspective, T+D+CT vs. CT was associated with an additional cost of $445, which gained an ICER of $5,167/QALY, and the T+D+CT vs. D+CT yielded cost savings of $2. At the WTP of $100,000, T+D+CT was considered cost-effective compared with CT but was not cost-effective compared with D+CT from the perspective of the healthcare sector. It was considered highly cost-effective compared with CT or D+CT from the societal perspective (Supplementary eTable S2).

Results of scenario analysis were consistent with the base case analysis (Supplementary eTable S2). Results of the short-term cost-effectiveness analysis did not support that T+D+CT was an economical treatment compared with CT from the perspective of the healthcare sector (Supplementary eTable S3). The subgroup analysis found that T+D+CT vs. CT or D+CT entailed lower incremental costs and higher incremental QALYs among patients with non-squamous mNSCLC than patients with squamous mNSCLC. The T+D+CT vs. CT remained cost-effective among patients with non-squamous mNSCLC from the perspective of the healthcare sector whilst being not cost-effective among patients with squamous mNSCLC (Supplementary eTable S4).

### 3.2 One-way sensitivity analyses

From the perspective of the US healthcare sector, the annual discount rate was the primary factor affecting ICER (Supplementary eFigure S2). For T+D+CT vs. CT, the model was also sensitive to the costs of subsequent immunotherapy, tremelimumab, palliative care and death, pemetrexed cost, and durvalumab, which altogether affected the cost-effectiveness of T+D+CT (Supplementary eFigure S2). If T+D+CT was cost-effective compared with CT, the annual discount rate should be controlled under 3.12%. Alternatively, treatment costs should be controlled under $9,746 for tremelimumab, under $3,970 for pemetrexed, or under $1,534 for durvalumab. When the cost subsequent immunotherapy was contained within $11,729 or that the cost of palliative care and death was contained within $15,072, the CT would become an economical option. Although the cost of tremelimumab, cost of palliative care, cost of death, PD utility, and PFS utility were the top five factors affecting the economics of T+D+CT vs. D+CT, none of them could make ICER lower than the WTP threshold of $100,000 (Supplementary eFigure S2). In addition, the cost-effectiveness of T+D+CT was associated with the period of receiving durvalumab monotherapy post-PD and it was considered cost-effective compared with CT if the period was more than 10 months. The T+D+CT had the lowest ICER compared with D+CT after receiving a four-month durvalumab monotherapy during PD, which was still over $100,000. From the societal perspective, all parameters were unlikely to change the cost-effectiveness of T+D+CT vs. CT or D+CT. The T+D+CT was always an economical option from the societal perspective, unbothered by any parameters.

### 3.3 Probabilistic sensitivity analysis

**FIGURE 2:**
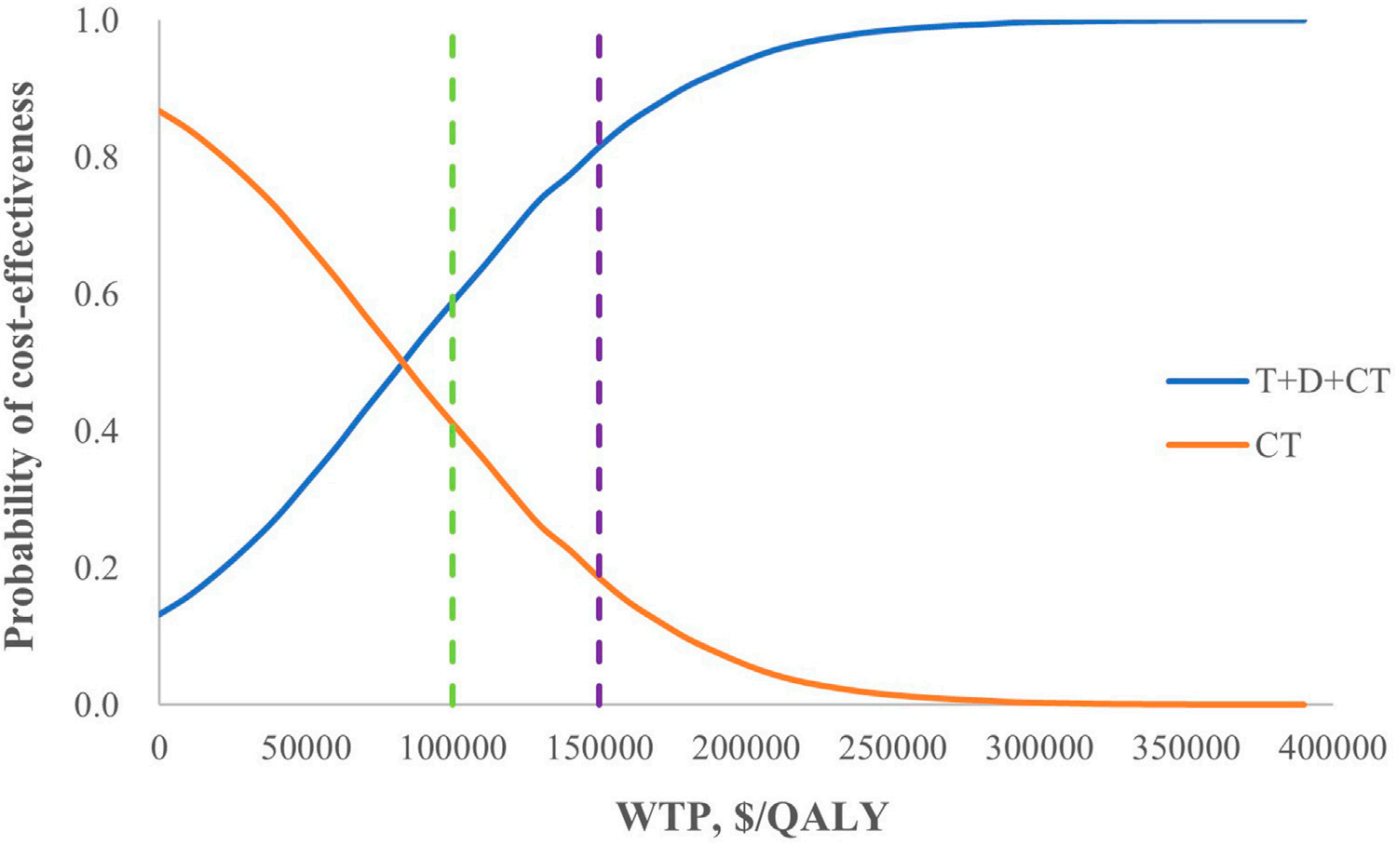
Cost-effectiveness acceptability curves for T+D+CT vs. CT from the perspective of the healthcare sector. T+D+CT, tremelimumab plus durvalumab and chemotherapy; CT, chemotherapy alone.

From the perspective of the healthcare sector, the probability of T+D+CT being cost-effective compared with CT was 59% at a threshold of $100,000/QALY and 82% at a threshold of $150,000/QALY (Figure 2), while it was only 0.04% compared with D+CT even if the WTP increased to $700,000/QALY (Supplementary eFigure S3). From the societal perspective, the probability of T+D+CT being cost-effective was 100% at the threshold of $100,000/QALY (Supplementary eFigure S3).

By changing the horizon of simulation time, it was found that the minimum value of ICERs for T+D+CT vs. CT and T+D+CT vs. D+CT were 13 years (Supplementary eFigure S4). Therefore, we calculated the EVPI of T+D+CT vs. CT or D+CT for a 13-year simulation time horizon. The EVPIs were estimated to be $1811.53 per patient for T+D+CT vs. CT, and $0.00 per patient for T+D+CT vs. D+CT.

## 4 Discussion

In this cost-effectiveness analysis, we found that T+D+CT could be considered cost-effective, compared with CT, as the first-line treatment for patients with mNSCLC, though this was not the case when T+D+CT was compared with D+CT. Our model for T+D+CT vs. CT was particularly sensitive to assumptions regarding the annual discount rate and treatment costs. The model for T+D+CT vs. D+CT was also sensitive to health utilities, but these assumptions did not change the cost-effectiveness of T+D+CT vs. D+CT. To our knowledge, this is the first study to evaluate the cost-effectiveness of fist-line T+D+CT for mNSCLC from the perspectives of the US healthcare sector and the society.

Pemetrexed and durvalumab were the main treatment options for the T+D+CT and D+CT arms during the maintenance phase, the costs of which were factors that, in this study, only the model of T+D+CT vs. CT was sensitive to. The shorter duration of pemetrexed and durvalumab in the short-term cost-effectiveness analysis did not show the economics of T+D+CT compared with CT, indicating that the duration of immunotherapy is also a factor affecting the economics of the treatment, but there is currently no clear definition of the duration of immunotherapy (Courtney et al., 2021). Both the models of T+D+CT vs. CT and T+D+CT vs. D+CT showed sensitivity to the cost of tremelimumab, which was only used in the T+D+CT arm. The proportions of patients with PD or death and patients receiving subsequent immunotherapy after PD in the CT arm were significantly higher than that in the T+D+CT and D+CT arms. Controlling the costs of subsequent immunotherapy and palliative care and death would help to reduce the treatment costs of the CT arm.

The sensitivity analysis showed that T+D+CT in patients who continued to receive durvalumab for 10 months or more after PD was cost-effective compared with CT. The phase-III ARCTIC (NCT02352948) trial reported that the median duration of response (DoR) among patients receiving T+D as the third-line treatment was 12.2 months, which was longer than that of the POSEIDON trial (9.5 months) and included the ten-month duration (Planchard et al., 2020). The subgroup analysis proved that histology was among factors affecting the economics of T+D+CT compared with CT and the median DoR of patients with non-squamous mNSCLC was significantly longer than that of patients with squamous mNSCLC in the T+D+CT arm (16.4 months vs. 5.6 months) (Johnson et al., 2023). The difference in the proportion of patients with non-squamous mNSCLC (63.31% in the POSEIDON trial vs. 75.86% in the ARCTIC trial) and the expression of PD-L1 (only patients with a tumor proportion score of 25% or more received T+D in the ARCTIC trial, patients with tumor proportion score less than 25% also received T+D+CT in the POSEIDON trial) may be partly responsible for the difference in the median DoR between POSEIDON and ARCTIC trials (Planchard et al., 2020; Johnson et al., 2023). At the WTP threshold of $100,000/QALY, patients could continue to receive durvalumab for 10 months or more after PD if they meet the criteria, which could be extended for patients scoring over 25% on tumor proportion or patients with non-squamous mNSCLC.

We explored the impact of uncertainties on decision-making by conducting probabilistic sensitivity analyses over 10,000 simulations. Based on our EVPI outcomes, when all uncertainties were considered and the best treatment option was identified for each individual patient, patients with mNSCLC in the US were projected to save a total of $601 million when eligible patients received T+D+CT, $53 million when eligible patients received D+CT, and $832 million when eligible patients received CT (Siegel et al., 2021).

Two earlier phase-III trials (MYSTIC and NEPTUNE) of T+D vs. CT as the first-line treatment for mNSCLC did not show any statistically significant improvement in OS between T+D and CT (Rizvi et al., 2020; de Castro et al., 2023). The MYSTIC trial (NCT02453282), conducted among mNSCLC patients with no sensitizing epidermal growth factor receptor (EGFR) mutation or anaplastic lymphoma kinase (ALK) genomic tumor aberrations, did not meet its primary end points of improved OS or PFS for T+D vs. CT in patients with over 25% tumor proportion score, but identified a tumor mutational burden from blood (bTMB) threshold of 20 mut/Mb for optimal OS benefit (Rizvi et al., 2020). The POSEIDON trial considered the bTMB ≥ 20 mut/Mb population when tested the secondary endpoint of OS for T+D+CT vs. CT after meeting the primary endpoints of OS and PFS benefits (Johnson et al., 2023). However, the NEPTUNE trial (NCT02453282) for mNSCLC with EGFR and ALK mutations missed its primary end point of improved OS for T+D vs. CT in patients with bTMB≥20 mut/Mb (de Castro et al., 2023).

Except for mNSCLC, durvalumab in combination with tremelimumab has been approved for patients with unresectable hepatocellular carcinoma (uHCC) in the US based on the HIMALAYA trial (NCT03298451) (Abou-Alfa et al., 2022). The indications of mNSCLC and uHCC were under regulatory review in several regions and countries worldwide, including Europe, Japan, Australia, Canada, and China (Keam, 2023). In addition, the evaluation for some other indications is also ongoing, though little supporting evidence has been generated. In this regard, the CASPIAN trial (NCT03043872) for extensive-stage small cell lung cancer showed that adding T+D to platinum-etoposide was not more effective than platinum-etoposide alone as the first-line treatment (Goldman et al., 2021). The DANUBE trial (NCT02516241) for unresectable, locally advanced or metastatic urothelial carcinoma showed that T+D was not more effective than CT as the first-line treatment (Powles et al., 2020). The phase-II trials for advanced biliary tract cancer, progressive, refractory, advanced thyroid carcinoma, cervical cancer, and tumor mutational burden-high and/or microsatellite instability-high of advanced solid tumors are also ongoing (Keam, 2023).

The positive clinical outcomes and cost-effectiveness data from our study can support providers in advocating for the inclusion of this combination therapy in treatment protocols. Providers can balance clinical efficacy with financial considerations to guide patients towards therapies that offer the best value. By presenting evidence of both the clinical benefits and the cost savings associated with this treatment, providers can make a stronger case for its adoption in clinical practice, potentially improving patient outcomes and reducing overall healthcare costs. For policy stakeholders, our findings offer valuable evidence to support policy discussions about including cost-effective treatments in formularies. Although cost-effectiveness is not typically the primary criterion for formulary decisions in the US, the growing emphasis on value-based care models could lead to greater consideration of economic evaluations. Our study can inform budget impact analyses, helping policymakers understand the long-term economic benefits of adopting durvalumab plus tremelimumab. Additionally, this evidence can influence reimbursement policies by highlighting the potential for cost savings and improved patient outcomes, encouraging the adoption of more cost-effective therapies through value-based reimbursement schemes. Future research should focus on gathering real-world evidence to validate the cost-effectiveness of this combination therapy in diverse patient populations. This can help address any discrepancies between clinical trial populations and routine care settings. Additionally, studies that specifically analyze the impact of cost-effectiveness evidence on formulary decisions and healthcare policies in the US can provide insights into how such evidence can be more effectively utilized in the decision-making process. By continuing to build on this foundation, researchers can contribute to a more comprehensive understanding of the value of new treatments in real-world settings, ultimately guiding better healthcare decisions and policy formulations.

## 5 Limitations

Our analysis has several limitations. First, the survival data and treatment strategies used in our model were only from one phase III randomized controlled trial (POSEIDON). The trial population may be slightly younger and healthier compared to the general population, potentially leading to differences in treatment tolerance and outcomes. Differences in income level and insurance type can affect access to treatment and adherence, potentially influencing real-world effectiveness. While efforts were made to include a diverse population, certain racial and ethnic groups might still be underrepresented, which could affect the generalizability of the findings. The results of more clinical studies could help to build a more robust prediction model. However, the other two published phase-III clinical studies did not meet the primary endpoint of OS (or PFS) benefits. Second, the health utilities and treatment costs used in this study were mainly derived from previous studies, whose research protocol and patient characteristics differed from those of the POSEIDON trial. Although the cost-effectiveness analysis from the perspective of the US healthcare sector showed that T+D+CT was cost-effective compared with CT and was not cost-effective compared with D+CT, the results of clinical trials conducted in individual patients or by other medical institutions may be different, as models were sensitive to assumptions of health utilities and treatment costs in the sensitivity analysis. Third, many alternative treatment options for mNSCLC were not assumed. However, the results may not be overturned by these unassumed parts as the proportion of patients who chose other treatments during the subsequent anticancer therapy in the POSEIDON trial was small and the ICERs of T+D+CT relative to CT and D+CT were far from the threshold of WTP. Fourth, this study did not take into account the impact of some factors related to the effectiveness on the economics of the treatment. The POSEIDON trial only considered two PD-L1 expression levels, 50% and 1%. If 25% was used as the cutoff, whether it would produce different economic results is unknown. In addition, the trial did not report the survival data of patients with bTMB ≥ 20 and bTMB < 20, whether the economic results were related to the bTMB level is also unknown.

## 6 Conclusion

This economic evaluation found that D+T+CT could be considered cost-effective if compared with CT alone but could not if compared with D+CT as the first-line treatment for patients with mNSCLC from the perspective of the US healthcare sector. However, these results only stood true in the non-squamous mNSCLC cohorts. The results of squamous mNSCLC cohorts did not support the economics of D+T+CT compared with CT alone. From the societal perspective, D+T+CT was cost-effective, independent of histology. Alongside improving patient survival, the duration and high-cost of immunotherapy are also issues to be considered by the healthcare sector (Durvalumab, 2023).

## Data Availability

The datasets presented in this study can be found in online repositories. The names of the repository/repositories and accession number(s) can be found in the article/Supplementary Material.

## Abbreviations

AWP, average wholesale price; BSC, best supportive care; CMS, Centers for Medicare and Medicaid Services; CT, chemotherapy; CTLA-4, cytotoxic T-lymphocyte associated protein 4; D+CT, durvalumab plus chemotherapy; DoR, duration of response; EVPI, expected value of perfect information; ICER, incremental cost-effectiveness ratio; mAb, monoclonal antibody; mNSCLC, metastatic non-small cell lung cancer; NSCLC, non-small cell lung cancer; OS, overall survival; OWSAs, one-way deterministic sensitivity analyses; PD, progressive disease; PD-L1/PD-1, programmed cell death ligand-1/programmed cell death-1; PFS, progression-free survival; PSAs, probabilistic sensitivity analyses; QALYs, quality-adjusted life years; QoL, quality of life; SDs, standard deviations; T+D+CT, durvalumab plus tremelimumab and chemotherapy; TRAEs, treatment-related adverse events; WTP, willingness-to-pay.
