# Supplementary material for: Cost-effectiveness of durvalumab plus tremelimumab in combination with chemotherapy for the treatment of metastatic non-small-cell lung cancer from the US healthcare sector’s and societal perspectives
Source: Front Pharmacol. 2024 Jun 10;15:1256992. doi: 10.3389/fphar.2024.1256992 (PMC11194367; doi:10.3389/fphar.2024.1256992)
Supplement: Supplementary file 1 [file DataSheet1.PDF]

Supplementary Material

Cost-effectiveness of Durvalumab plus Tremelimumab in Combination with Chemotherapy for the Treatment of Metastatic Non-Small-Cell Lung Cancer from the US Healthcare Sector's and Societal Perspectives

Yena Gan[1], Fenghao Shi[2,3], He Zhu[2,3], Huangqianyu Li[2,3], Sheng Han[2,3*], Duoduo Li[1*]

[1]Dongzhimen Hospital, Beijing University of Chinese Medicine, Beijing, China

[2]International Research Center for Medicinal Administration, Peking University, Beijing, China

[3]School of Pharmaceutical Sciences, Peking University, Beijing, China

*** Correspondence:**
Sheng Han
hansheng@bjmu.edu.cn

Duoduo Li
tarako@163.com

eMethods

1 Validation of Parametric Survival Functions

The Kaplan-Meier (K-M) curves of overall survival (OS) and progression-free disease (PFS) were digitalized by WebPlotDigitizer (version 4.5) and were used to estimate the individual-patient level data (IPD) points using the R software (version 4.1.3). Parametric survival functions were derived from IPD, which were used to accurately estimate the survival benefit over 15 years, while in the absence of IPD, we calibrated parametric survival functions using the R software[1]. The following regression models were usually used to construct the parametric survival functions: Exponential, Gamma, Gompertz, Weibull, Log-Logistics, Log-Normal, Generalized Gamma. The Akaike's Information Criterion (AIC) and Bayesian Information Criterion (BIC) values were compared and their lowest sum were used to choose the best-fit parametric survival function (**eTable 1**). When the selected parametric survival

function with the lowest sum did not align with the published K-M curve, we chose the function based on the lowest BIC value.

2 Methods for Determining Drug Costs

We constructed a hypothetical cohort of patients with characteristics consistent with those in the POSEIDON clinical trial. The patients had stage-IV NSCLC, a Eastern Cooperative Oncology Group performance status of 0 or 1, and were with no sensitizing epidermal growth factor receptor (EGFR) mutation or anaplastic lymphoma kinase (ALK) genomic tumor aberrations[2].

Per the POSEIDON trial[2], patients in the tremelimumab plus durvalumab and chemotherapy (T+D+CT) arm received durvalumab at a dose of 1,500 mg plus tremelimumab at a dose of 75mg and chemotherapy every three weeks for up to four 21-day cycles, followed by durvalumab 1,500 mg once every four weeks until progressive disease (PD), with one additional tremelimumab dose after chemotherapy at week 16. In the durvalumab plus chemotherapy (D+CT) arm, patients received durvalumab 1,500 mg plus chemotherapy every three weeks for up to four 21-day cycles, followed by durvalumab 1,500 mg once every four weeks until PD. In the chemotherapy arm, patients received chemotherapy every three weeks for up to six cycles. All patients could receive carboplatin plus nab-paclitaxel during chemotherapy, regardless of histology. For patients with squamous histology, treatment with cisplatin or carboplatin plus gemcitabine was another option, whilst that for patients with nonsquamous histology was cisplatin or carboplatin plus pemetrexed. Therefore, with our costs of chemotherapy, we calculated a weighted average based on the proportion of two tumor histologic types and three chemotherapeutic agents reported in the trial.

A number of assumptions were made for this analysis. As the period of durvalumab monotherapy after PD was not specified, we assumed that patients who experienced PD continued to receive durvalumab monotherapy for up to two years. We also conducted the period of receiving durvalumab monotherapy post-PD in the sensitive analysis. After patients stopped the first-line treatment, they continued to receive subsequent anticancer therapy until the data collection point, with their therapeutic options including immunotherapy, cytotoxic chemotherapy, radiotherapy, and best supportive care (BSC). The proportion of patients receiving subsequent anticancer therapy was consistent with POSEIDON. Later, patients in all groups received BSC until one month before death, when they switched to receive palliative care. The specific treatment regimens for immunotherapy and cytotoxic chemotherapy were not reported in the POSEIDON trial. We assumed that immunotherapy was pembrolizumab or atezolizumab, single-agent cytotoxic chemotherapy was docetaxel, and platinum-doublet cytotoxic chemotherapy was cisplatin or carboplatin[3]. The effect of the proportion of patients receiving pembrolizumab or atezolizumab and

cisplatin or carboplatin on the cost of subsequent immunotherapy and chemotherapy was considered in sensitivity analysis. We did not include “targeted therapy” and “other systemic therapy” in subsequent anticancer therapy because those agents were not specified. The costs of each cycle were calculated in accordance with the POSEIDON trial, which provided a three-week treatment period during the chemotherapy phase and a four-week treatment period during the maintenance phase.

3 Methods for Sensitivity Analysis

The costs and QALYs were related to the simulation time horizon. We restricted the simulation time horizon to five-year to examine the short-term cost-effectiveness of T+D+CT and analyzed incremental cost-effectiveness ratio (ICER) under different horizons during 5-15 years. The expected value of perfect information was analyzed at the simulation time horizon with the lowest ICERs.

4 Methods for Subgroup Analysis

According to the histology of mNSCLC, patients were divided into two groups, squamous and nonsquamous mNSCLC. Survival data were derived from the POSEIDON clinical trial. Except for the chemotherapy regimen, treatments were consistent with the basic case model. For patients with squamous mNSCLC, they received either cisplatin/carboplatin plus gemcitabine or carboplatin plus nab-paclitaxel in their chemotherapy phase. For patients with nonsquamous mNSCLC, they received either cisplatin/carboplatin plus pemetrexed or carboplatin plus nab-paclitaxel in the chemotherapy phase. Judging from the data reported in the POSEIDON trial, some patients in the subgroups did not receive the above-mentioned chemotherapy regimens and the possibility of quitting chemotherapy regimens actively or passively (treatment-related adverse events, TRAEs) cannot be ruled out. It was assumed that the reported number of patients receiving gemcitabine or pemetrexed was the actual number and other patients received carboplatin plus nab-paclitaxel. Differences in the incidence of TRAEs between the two subgroups were not specifically described in the trial and it was thus assumed that their distribution between two subgroups was consistent with the base case model. The PFS and PD utilities of the two subgroups were derived from previous studies[4-6] and the difference in the disutility of TRAEs was not deliberately distinguished.

eTable 1 Statistical fits for parametric survival functions

Parametric survival function	OS (CT)						PFS (CT)					
	mNSCLC		nonsquamous mNSCLC		squamous mNSCLC		mNSCLC		nonsquamous mNSCLC		squamous mNSCLC	
	AIC	BIC	AIC	BIC	AIC	BIC	AIC	BIC	AIC	BIC	AIC	BIC

Exponential	2151.30	2155.12	**1367.38**	1364.01	786.63	**789.43**	1492.69	1496.51	955.49	958.86	534.77	537.57
Gamma	2149.34	2156.98	1370.41	1363.68	787.43	793.04	1454.08	1461.72	944.23	950.96	504.79	510.40
Gompertz	2153.25	2160.89	1372.73	1366.00	788.51	794.12	1488.64	1496.28	956.11	962.85	508.57	514.18
Weibull	2151.06	2158.70	1371.22	1364.49	788.07	793.67	1463.33	1470.97	947.14	953.87	**503.13**	**508.74**
Log-Logistics	2145.50	**2153.14**	1369.75	**1363.02**	787.06	792.67	**1444.20**	**1451.84**	**939.11**	**945.84**	514.60	520.21
Log-Normal	2149.27	2156.91	1374.04	1367.31	**785.99**	791.59	1446.16	1453.80	948.43	955.16	513.85	519.45
Generalized Gamma	**2144.98**	2156.44	1373.53	1363.43	786.30	794.71	1446.69	1458.15	943.92	954.01	505.03	513.44

Parametric survival function	OS (D+CT)						PFS (D+CT)					
	mNSCLC		nonsquamous mNSCLC		squamous mNSCLC		mNSCLC		nonsquamous mNSCLC		squamous mNSCLC	
	AIC	BIC	AIC	BIC	AIC	BIC	AIC	BIC	AIC	BIC	AIC	BIC
Exponential	2099.70	2103.52	1281.44	1284.78	**811.40**	**814.25**	1600.24	1604.06	979.55	982.89	627.73	630.59
Gamma	2101.67	2109.31	1283.04	1289.73	812.86	818.56	1594.35	1601.99	980.70	987.39	**620.32**	**626.02**
Gompertz	2097.23	2104.88	1279.46	1286.14	813.38	819.09	1601.24	1608.88	977.40	984.08	626.87	632.57
Weibull	2101.58	2109.22	1282.48	1289.16	812.97	818.68	1598.33	1605.98	981.47	988.16	621.20	626.90
Log-Logistics	**2090.69**	**2098.34**	**1277.76**	**1284.44**	814.85	820.55	**1573.99**	**1581.63**	966.89	973.58	622.24	627.94
Log-Normal	2096.61	2104.26	1279.62	1286.30	824.99	830.70	1575.81	1583.46	**965.72**	**972.41**	631.14	636.84
Generalized Gamma	2094.45	2105.92	1279.69	1289.72	814.78	823.33	1577.77	1589.24	967.40	977.43	622.26	630.82

Parametric survival function	OS (T+D+CT)						PFS (T+D+CT)					
	mNSCLC		nonsquamous mNSCLC		squamous mNSCLC		mNSCLC		nonsquamous mNSCLC		squamous mNSCLC	
	AIC	BIC	AIC	BIC	AIC	BIC	AIC	BIC	AIC	BIC	AIC	BIC
Exponential	2052.47	2056.29	1241.10	1244.46	790.46	793.28	1566.07	1569.89	946.55	949.91	575.21	578.03

Gamma	2054.46	2062.11	1242.64	1249.37	791.53	797.17	1561.77	1569.41	946.59	953.32	564.40	570.04
Gompertz	2044.45	2052.10	1236.52	1243.25	790.82	796.46	1565.59	1573.24	946.57	953.30	576.69	582.33
Weibull	2054.05	2061.70	1241.88	1248.61	792.23	797.87	1565.37	1573.02	947.87	954.60	568.34	573.98
Log-Logistics	2033.73	2041.38	1234.12	1240.85	**784.31**	**789.95**	**1538.51**	**1546.15**	935.31	942.04	**557.02**	**562.66**
Log-Normal	**2032.46**	**2040.10**	**1233.07**	**1239.80**	788.66	794.30	1543.81	1551.45	**934.49**	**941.22**	559.86	565.50
Generalized Gamma	2034.38	2045.85	1235.05	1245.14	788.85	797.31	1545.37	1556.84	936.35	946.45	561.32	569.78

CT, chemotherapy alone; D+CT, durvalumab plus chemotherapy; T+D+CT, tremelimumab plus durvalumab and chemotherapy; mNSCLC, metastatic non-small-cell lung cancer; AIC, Akaike's Information Criterion; BIC, Bayesian Information Criterion; PFS, progression-free survival; OS, overall survival.

eTable 2 Results of base case and scenario analyses for patients with mNSCLC from the US health care sector and social perspectives

**Analysis Perspective**		**Cost, $**	**LYs**	**QALYs**	**Incremental Costs, $**	**Incremental QALYs**	**ICER/QALY**
**Base Case Analysis**	US health care sector perspective						
	T+D+CT	368,076	1.50	0.55	7,108	0.09	82,501
	CT	360,968	1.13	0.46			
	T+D+CT	368,076	1.50	0.55	27,779	0.02	1,243,868
	D+CT	340,297	1.40	0.53			
	US social perspective						
	T+D+CT	68,079	1.50	0.55	445	0.09	5,167

	CT	67,634	1.13	0.46			
	T+D+CT	68,079	1.50	0.55	-2	0.02	-84
	D+CT	68,081	1.40	0.53			
	US health care sector perspective						
	T+D+CT	367,501	1.42	0.55	6,770	0.09	78,383
	CT	360,732	1.08	0.46			
	T+D+CT	367,501	1.42	0.55	26,967	0.02	1,151,759
	D+CT	340,534	1.27	0.53			
**Scenario Analysis**	US social perspective						
	T+D+CT	69,587	1.42	0.55	8,041	0.09	93,104
	CT	61,546	1.08	0.46			
	T+D+CT	69,587	1.42	0.55	-173	0.02	-7,398
	D+CT	69,760	1.27	0.53			

mNSCLC, metastatic non-small cell lung cancer; T+D+CT, tremelimumab plus durvalumab and chemotherapy; D+CT, durvalumab plus chemotherapy; CT, chemotherapy alone; LYs, life years; QALYs, quality-adjusted life years; ICER, incremental cost-effectiveness ratio.

eTable 3 Results of short-term (5-year) cost-effectiveness analysis for patients with mNSCLC from the US health care sector and social perspectives

Analysis Perspective	Cost, $	LYs	QALYs	Incremental Costs, $	Incremental QALYs	ICER/QALY
US health care sector perspective						
T+D+CT	302,326	1.15	0.54	9,814	0.08	121,414
CT	292,512	0.94	0.46			
T+D+CT	302,326	1.15	0.54	28,564	0.02	1,358,186
D+CT	273,762	1.07	0.52			
US social perspective						
T+D+CT	58,604	1.15	0.54	451	0.08	5,580
CT	58,153	0.94	0.46			
T+D+CT	58,604	1.15	0.54	-2	0.02	-95
D+CT	58,606	1.07	0.52			

mNSCLC, metastatic non-small cell lung cancer; T+D+CT, tremelimumab plus durvalumab and chemotherapy; D+CT, durvalumab plus chemotherapy; CT, chemotherapy alone; LYs, life years; QALYs, quality-adjusted life years; ICER, incremental cost-effectiveness ratio.

eTable 4 Results of subgroup analysis for patients with nonsquamous or squamous mNSCLC from the US health care sector and social perspectives

Analysis Perspective		Cost, $	LYs	QALYs	Incremental Costs, $	Incremental QALYs	ICER/QALY
Nonsquamous mNSCLC	US health care sector perspective						
	T+D+CT	353,964	1.93	0.69	162	0.13	1,266
	CT	353,802	1.25	0.57			
	T+D+CT	353,964	1.93	0.69	19,478	0.06	353,952
	D+CT	334,486	1.62	0.64			
	US social perspective						
	T+D+CT	68,973	1.93	0.69	-607	0.13	-4,728
	CT	69,579	1.25	0.57			
	T+D+CT	68,973	1.93	0.69	-1,442	0.06	-26,205
	D+CT	70,415	1.62	0.64			
Squamous mNSCLC	US health care sector perspective						
	T+D+CT	398,094	1.00	0.35	15,534	0.05	314,248
	CT	382,560	0.76	0.30			
	T+D+CT	398,094	1.00	0.35	38,746	0.00	27,456,782

D+CT	359,348	0.87	0.35			
US social perspective						
T+D+CT	70,408	1.00	0.35	839	0.05	16,962
CT	69,569	0.76	0.30			
T+D+CT	70,408	1.00	0.35	-7	0.00	-4,639
D+CT	70,415	0.87	0.35			

mNSCLC, metastatic non-small cell lung cancer; T+D+CT, tremelimumab plus durvalumab and chemotherapy; D+CT, durvalumab plus chemotherapy; CT, chemotherapy alone; LYs, life years; QALYs, quality-adjusted life years; ICER, incremental cost-effectiveness ratio.

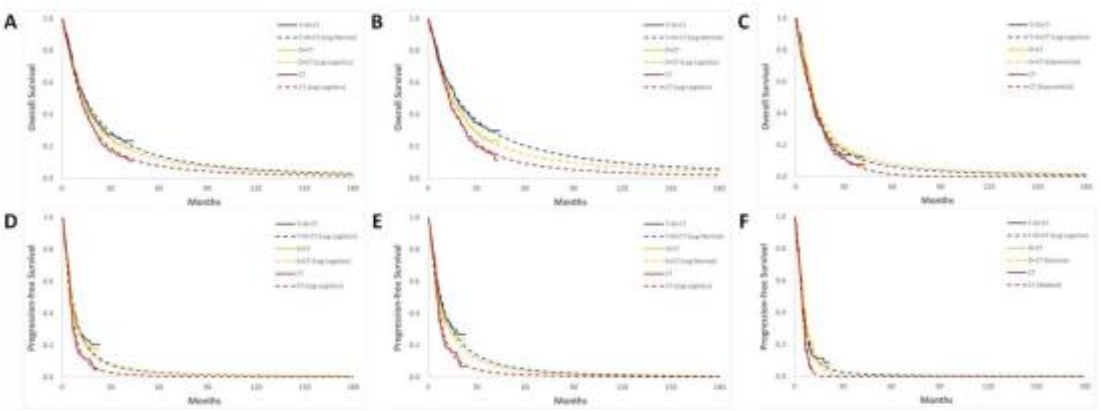

eFigure 1 Standard and extended parametric survival curves for all patients with mNSCLC and patients with nonsquamous or squamous histology. (A) OS curves for all patients with mNSCLC. (B) OS curves for patients with nonsquamous mNSCLC. (C) OS curves for patients with squamous mNSCLC. (D) PFS curves for all patients with mNSCLC. (E) PFS curves for patients with nonsquamous mNSCLC. (F) PFS curves for patients with squamous mNSCLC. mNSCLC, metastatic non-small cell lung cancer; OS, overall survival; PFS, progression-free disease.

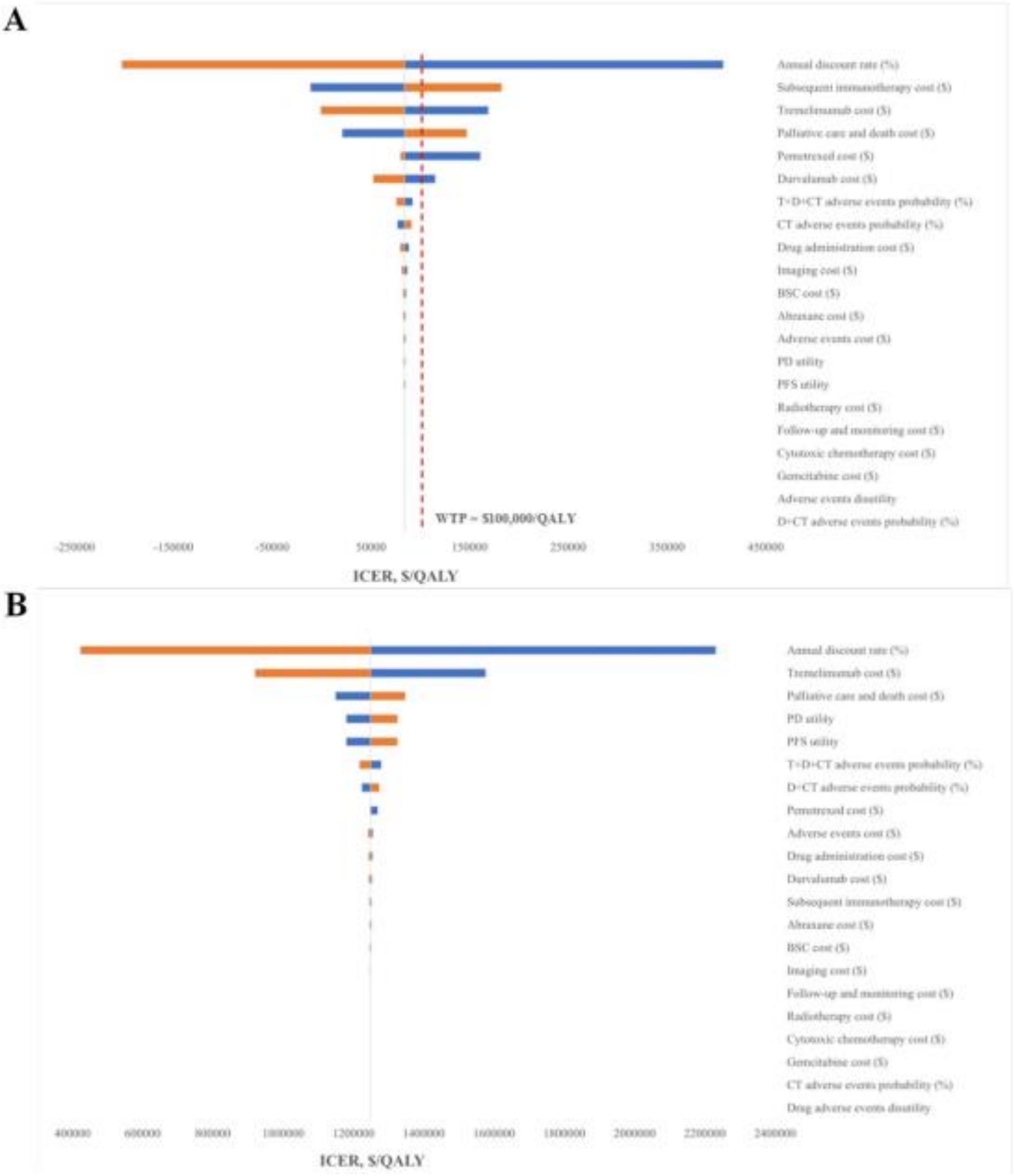

eFigure 2 Tornado diagrams of one-way sensitivity analysis for T+D+CT vs CT and T+D+CT vs D+CT from the US health care sector perspective. (A) Tornado diagram for T+D+CT vs CT. (B) Tornado diagram for T+D+CT vs D+CT. T+D+CT, tremelimumab plus durvalumab and chemotherapy; D+CT, durvalumab plus chemotherapy; CT, chemotherapy alone; PD, progressive disease; PFS, progression-free disease; BSC, best supportive care.

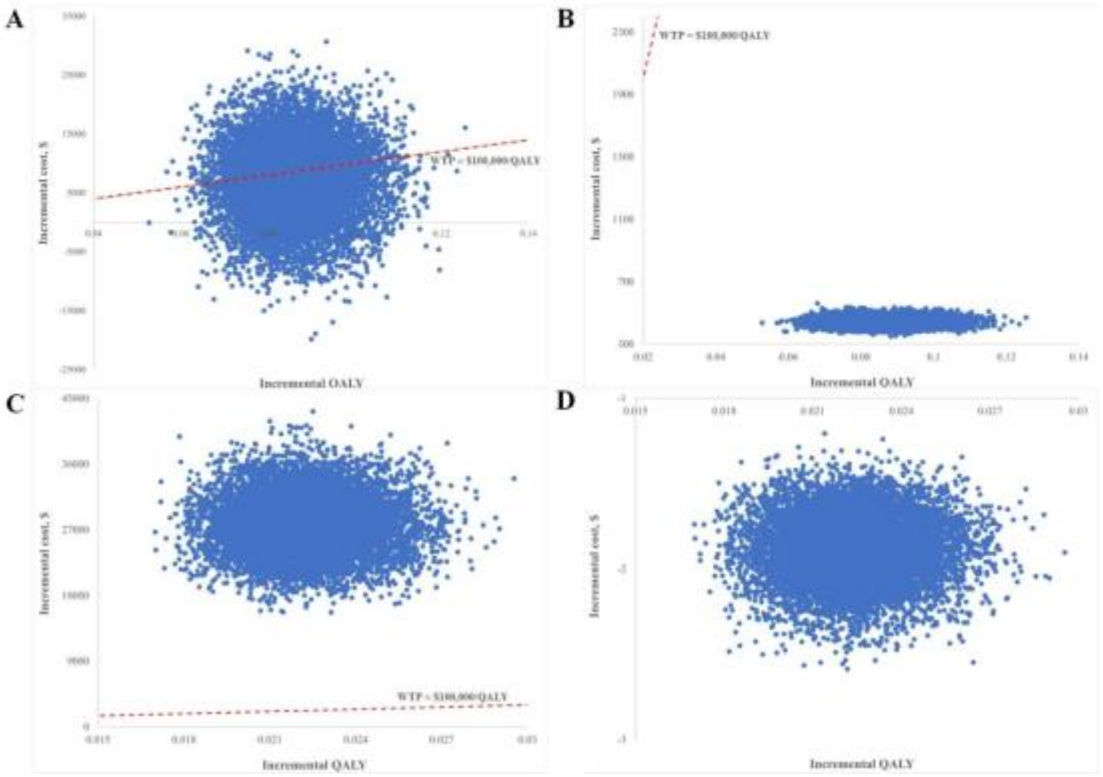

eFigure 3 Scatterplots of costs and effectiveness outcomes from a probabilistic sensitivity analysis through 10,000 Simulations. (A) Scatterplot for T+D+CT vs CT from the US health care sector perspective. (B) Scatterplot for T+D+CT vs CT from the US social perspective. (C) Scatterplot for T+D+CT vs D+CT from the US health care sector perspective. (D) Scatterplot for T+D+CT vs D+CT from the US social perspective. T+D+CT, tremelimumab plus durvalumab and chemotherapy; D+CT, durvalumab plus chemotherapy; CT, chemotherapy alone; QALY, quality-adjusted life year; WTP, willingness-to-pay.

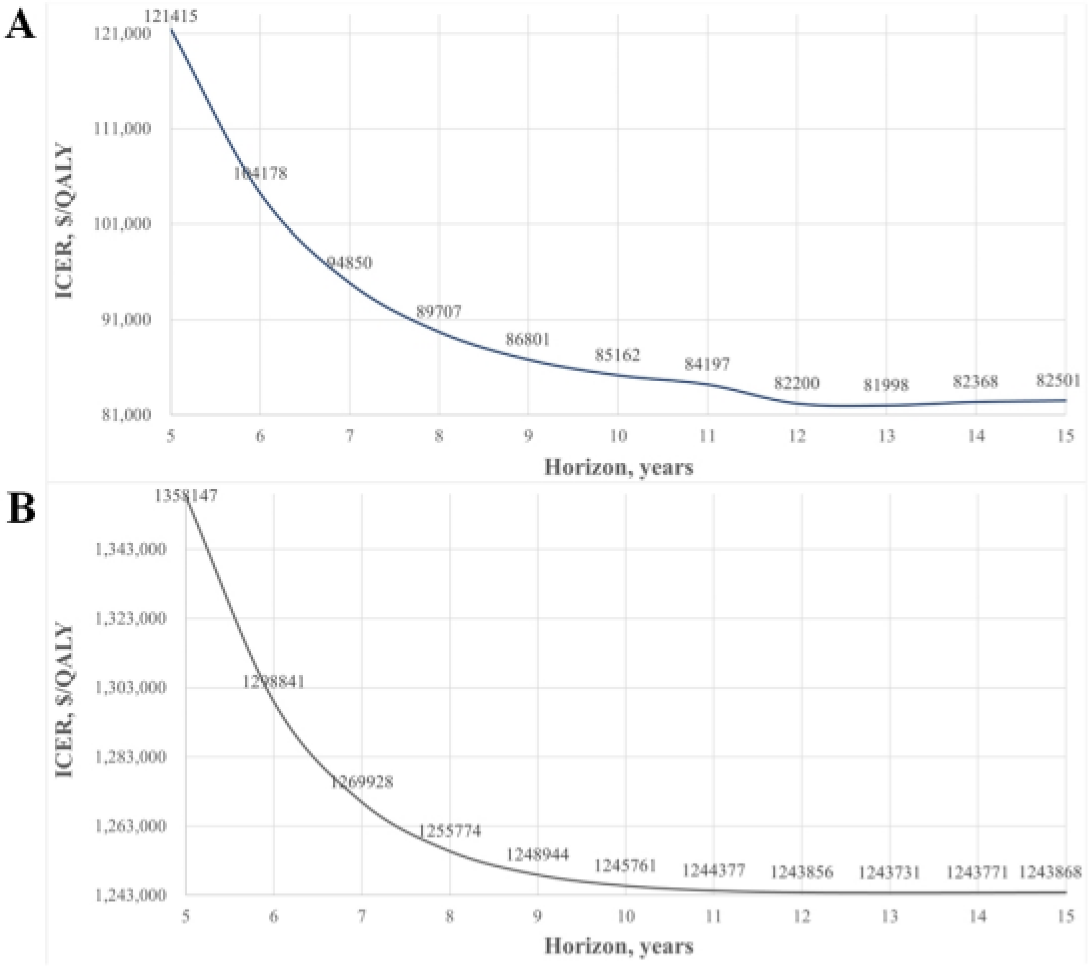

eFigure 4 ICERs for T+D+CT vs CT and T+D+CT vs D+CT during 5-15 years of simulation time horizon from the health care sector perspective. (A) ICER for T+D+CT vs CT. (B) ICER for T+D+CT vs D+CT. T+D+CT, tremelimumab plus durvalumab and chemotherapy; D+CT, durvalumab plus chemotherapy; CT, chemotherapy alone; QALY, quality-adjusted life year; ICER, incremental cost-effectiveness ratio.

eReferences

1. Choe JH, Abdel-Azim H, Padula WV, et al. Cost-effectiveness of Axicabtagene Ciloleucel and Tisagenlecleucel as Second-line or Later Therapy in Relapsed or Refractory Diffuse Large B-Cell Lymphoma. JAMA network open. 2022;5:e2245956. https://doi.org/10.1001/jamanetworkopen.2022.45956.

2. Johnson ML, Cho BC, Luft A, et al. Durvalumab With or Without Tremelimumab in Combination With Chemotherapy as First-Line Therapy for Metastatic Non-Small-Cell Lung Cancer: The Phase III POSEIDON Study. Journal of clinical oncology : official journal of the

American Society of Clinical Oncology. 2023;41:1213-1227. https://doi.org/10.1200/jco.22.00975.

3. Hellmann MD, Paz-Ares L, Bernabe Caro R, et al. Nivolumab plus Ipilimumab in Advanced Non-Small-Cell Lung Cancer. The New England journal of medicine. 2019;381:2020-2031. https://doi.org/10.1056/NEJMoa1910231.

4. Nafees B, Lloyd AJ, Dewilde S, et al. Health state utilities in non-small cell lung cancer: An international study. Asia-Pacific journal of clinical oncology. 2017;13:e195-e203. https://doi.org/10.1111/ajco.12477.

5. Chouaid C, Agulnik J, Goker E, et al. Health-related quality of life and utility in patients with advanced non-small-cell lung cancer: a prospective cross-sectional patient survey in a real-world setting. Journal of thoracic oncology : official publication of the International Association for the Study of Lung Cancer. 2013;8:997-1003. https://doi.org/10.1097/JTO.0b013e318299243b.

6. Nafees B, Stafford M, Gavriel S, et al. Health state utilities for non small cell lung cancer. Health and quality of life outcomes. 2008;6:84. https://doi.org/10.1186/1477-7525-6-84.
